# Annual Impervious Surface Data from 2001–2020 for West African Countries: Ghana, Togo, Benin and Nigeria

**DOI:** 10.1038/s41597-024-03610-8

**Published:** 2024-07-18

**Authors:** Andrews Korah, Michael C. Wimberly

**Affiliations:** Department of Geography and Environmental Sustainability, University of Oklahoma, Norman, OK 73019 USA

**Keywords:** Research data, Developing world, Geography

## Abstract

Impervious surface data are increasingly important for research and planning. Despite the availability of global and local urban land cover maps, regional data are lacking in Africa. We generated annual 30 m impervious cover data from 2001–2020 for Ghana, Togo, Benin, and Nigeria using the Landsat archive. We used random forest to predict impervious cover using 11 spectral indices and applied pixel-level temporal segmentation with the LandTrendr algorithm. Processing with LandTrendr improved the accuracy of the random forest predictions, with higher predicted-observed r^2^ (0.81), and lower mean error (−0.03), mean absolute error (5.73%), and root mean squared error (9.93%). We classified pixels >20% impervious as developed and < = 20% impervious as undeveloped. This classification had 93% overall accuracy and similar producer’s (79%) and user’s (80%) accuracies for developed area. Our maps had higher accuracy and captured more developed areas than comparable global datasets. This is the first regionally calibrated 30 m resolution impervious dataset in West Africa, which can support research on drivers and impacts of urban expansion and planning for future growth.

## Background & Summary

Globally, the number of people living in cities is increasing rapidly, with most of this growth occurring in urbanizing Asia and Africa^1–3^. Future projections are that Africa alone will add 930 million urban residents by 2050^4^. The growth of urban populations results in the expansion of impervious surfaces, including building roofs, glass, concrete, asphalt, and paved roads. These increases in developed areas have altered many of Earth’s surface processes, including energy balance, hydrological cycle, atmospheric circulation, and phenology^5^. These changes manifest through increased heat stress, urban fires, and floods combined with losses of cropland, natural habitats, and biodiversity^6–10^. With increasing urbanization in low-income countries, urban impervious surface data is needed to support governments, planners, and policymakers in managing the resulting impacts of impervious surface expansion. However, there is currently a lack of data on urban land cover change with 30 m or higher spatial resolution, broad-scale regional coverage, and consistent thematic content for Africa^11^.

Satellite Earth observations are increasingly used to develop data products for monitoring and evaluating urban expansion at global, regional, and local scales over a range of temporal and spatial resolutions. Commonly used data sources include daytime optical imagery, nighttime lights, and synthetic aperture radar from missions such as MODIS, VIIRS, Landsat, and Sentinel 1 and 2^12–17^. Several global data products are available, including Global Impervious Surface Area^18^, Global Human Settlement Layers^19,20^, Global Man-Made Impervious Surface^21^, Global Human Built-up and Settlement Extent^22^, Multi-temporal Global Impervious Surfaces^23^, Global 30-m Impervious Surface Map^24,25^, and the Global Artificial Impervious Area^26^. Although these global urban change datasets are often used for assessments at continental, national, and landscape scales, their local accuracies can be highly variable and are usually unknown^9^. Most of them are only available for a few time points separated by periods of 5–10 years, and none fully encompasses the numerous small cities that are critical components of the urban system. There are also a variety of national and local level urban land cover datasets throughout Africa, but differences in classification schemes and inconsistent processing methods hinder their usage across countries and cities^11,27^. Many of the impacts of global change manifest at the regional level; therefore, planning and policy interventions need to respond at this scale^9^. Thus, regionally optimized and consistent urban cover data are needed to support large-scale research, regional planning, future projections, and assessment of urban expansion impacts across multiple study areas.

Advances in machine learning and change detection algorithms create opportunities for improved regional land use and land cover monitoring compared with traditional classifiers such as maximum likelihood, minimum distance, and k-means^28–30^. Newer machine learning techniques such as support vector machines, neural networks, and random forests are more robust and computationally efficient, resulting in higher accuracy when used for land cover mapping^31–33^. Additionally, time series algorithms applied at the individual pixel level, including breaks for additive season and trend (BFAST)^34^, continuous change detection and classification (CCDC)^30^, and Landsat-based detection of Trends and Recovery (LandTrendr)^35–37^, can improve land cover change detection. BFAST and CCDC detect land cover change as deviations from long-term and seasonal trends using harmonic regression models, whereas LandTrendr estimates change trajectories by fitting segmented linear regression models to annual data.

Open access to the Landsat archive combined with machine learning and time series algorithms through the cloud-based Google Earth Engine (GEE) platform have revolutionized urban impervious surface cover mapping over large areas^23,35,38,39^. Studies in North America and Asia have leveraged the GEE cloud computing platform to generate regional impervious cover datasets^30,37,40,41^. However, there are currently no regionally calibrated and publicly available historical impervious surface data for West Africa, which is one of the fastest urbanizing regions in the world^11^. A major reason is limited internet resources, including unstable and slow bandwidth that limits the processing of thousands of images over large areas^42^. Impervious surface data that reflect neighborhood patterns and capture annual growth are particularly needed to support future projections and regional assessments of urban change impacts on people and the environment.

We addressed this gap by developing an accurate and consistent 20-year time series of continuous impervious surface data from 2001–2020 at a 30 m grid cell size using all available Landsat images. These data covered four heavily urbanized countries in West Africa: Ghana, Togo, Benin and Nigeria. In addition, we classified developed areas based on an impervious surface threshold and generated a separate product of their change over time. We carried out a comprehensive validation of both datasets to quantify their accuracies and compare them with currently available global impervious surface datasets. Our data products have been made available as TIFF images that can be used in standard software for geospatial data processing, and the code is available for updating the datasets and refining and extending the approach to new areas.

## Materials and Methods

### Study area

The coverage is 1.3 million square kilometers across four West African countries, consisting of Ghana, Togo, Benin, and Nigeria. These countries contain 73% of the 165 million urban population in West Africa and are rapidly expanding their developed area, with an annual urban expansion rate of 4.70% in Nigeria, 4.46% in Ghana, 4.19% in Benin, and 3.89% in Togo from 2001–2020^43–45^. Most (70%) of the cities in West Africa are in these four countries, with 1231 in Nigeria, 209 in Ghana, 110 in Benin, and 53 in Togo. Within these countries, natural population increase, rural-urban migrations, reclassification of rural areas into urban centers, uneven distribution of resources, and ethnic and political conditions increase demand for housing and other urban infrastructure and drive high rates of urban expansion^46,47^.

**Fig. 1:**
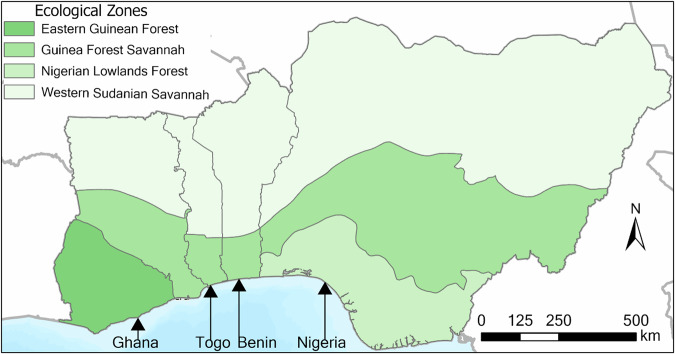
Study area countries and ecological zones. These ecological zones were generated by merging smaller terrestrial ecoregions into larger ones based on proximity and similar biophysical characteristics.

Each country has several ecological zones with different climates, soil moisture, vegetation, and land cover patterns (Fig. 1). The Western Sudanian Savannah (WSS) mainly consists of grasslands with short, dispersed trees with 600 mm to 1000 mm precipitation annually, resulting in semi-arid conditions during the dry season. The Guinea Forest Savannah (GFS) is a transitional zone with a mixture of dense tree cover and open grassland. This zone mostly receives 1600 to 2000 mm per year. The Eastern Guinea Forest (EGF) and Nigerian Lowlands Forest (NLF) receive more precipitation, ranging from 1500 mm to 2500 mm per year, and support dense tropical forests.

### Technical framework

**Fig. 2:**
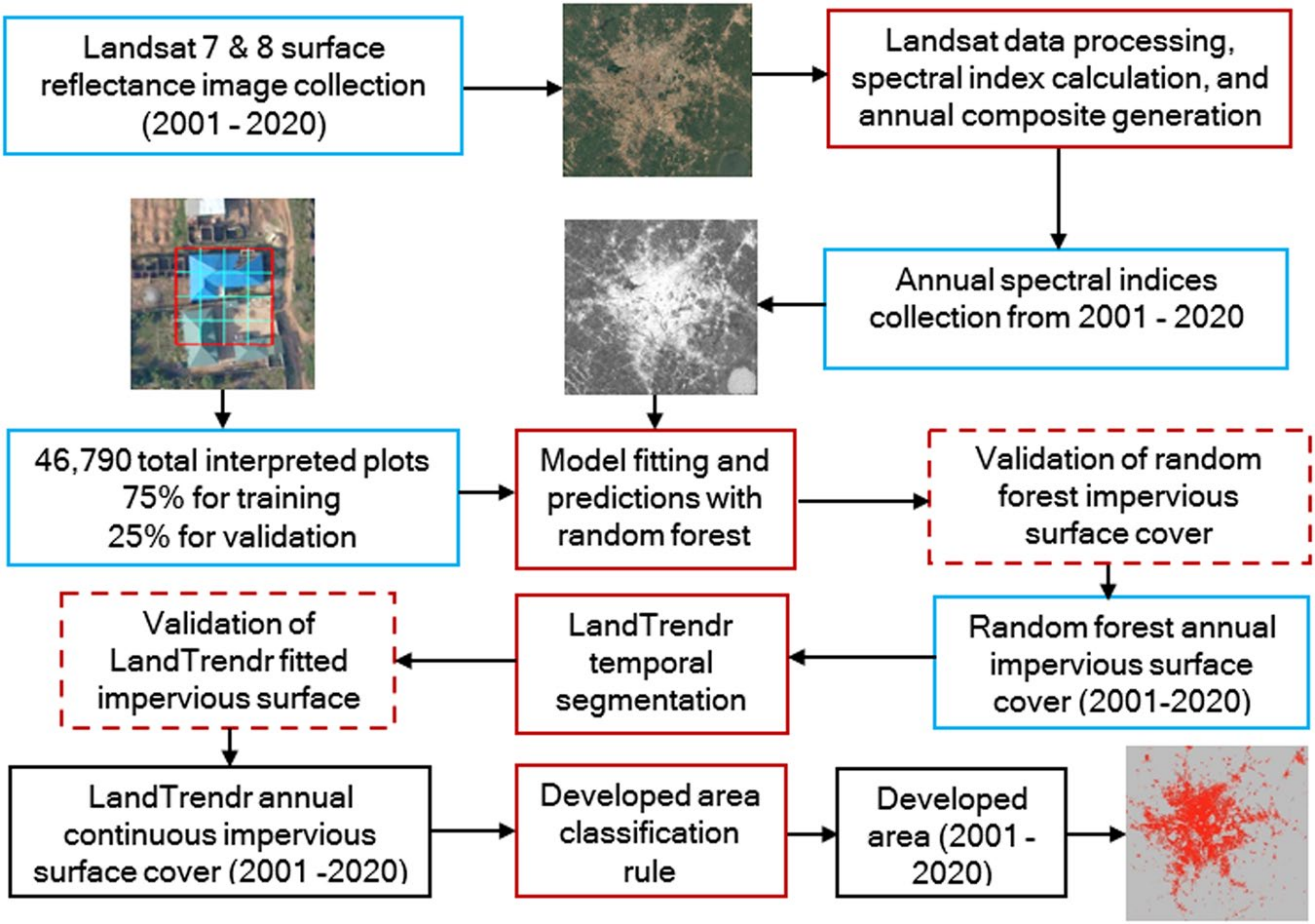
Technical workflow for generating the impervious cover dataset. The data at each stage is bounded in blue, processing methods in red, validation in dashed red, and final outputs in black.

The workflow for generating the impervious cover dataset is shown in Fig. 2. Step one involves processing Landsat imagery to remove clouds and cloud shadows and generating annual composites of spectral indices from 2001–2020. Step two is training random forest models with impervious surface observations from very high-resolution images and using them to predict impervious cover with the Landsat imagery. Step three is temporally segmenting the impervious cover time series for each pixel with LandTrendr. Step four involves classifying the LandTrendr processed impervious cover into developed and undeveloped areas. The following sections describe in detail the major workflow steps.

### Landsat data

**Fig. 3:**
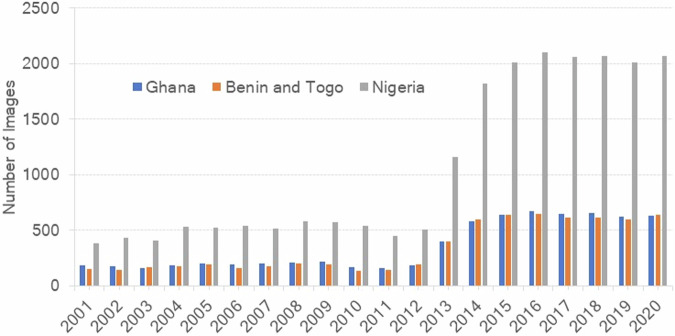
Availability of Landsat 7 and 8 images from 2001–2020. Each bar shows the number of images in each year for Ghana in blue, Benin and Togo in orange and Nigeria in gray.

We used Landsat 7 and 8 Collection 2 level-2 surface reflectance science products from 2001–2020. There was a total of 35065 images, including 7051 in Ghana, 6755 in Benin and Togo, and 21259 in Nigeria (Fig. 3). There was a mean of 850 Landsat 7 images per year from 2001–2012, and the addition of Landsat 8 increased the mean to 3110 images per year since 2013 (Fig. 3). We masked all clouds and cloud shadows using the pixel-quality layer with the C-function of the mask algorithm (CFMASK)^48,49^.

### Spectral indices and annual composites

Due to varying phenological and biophysical conditions across ecological regions, we used a combination of built-up, vegetation, burned area, and soil-based indices. Because the spectral properties of land surfaces vary geographically, we used diverse indices to minimize background noise and reduce errors. Previous comparisons of LandTrendr time series models found that using more than eight explanatory variables in land cover predictions resulted in lower errors than models based on fewer explanatory variables^50^. We computed 11 spectral indices (Table 1). They included 8 impervious surface indices: built-up area extraction index (BAEI)^51^, biophysical composition index (BCI)^52^, band ratio for built-up area index (BRBA)^53^, combinational biophysical composition index (CBCI)^54^, CBCI), normalized built-up area index (NBAI)^53^, normalized difference built-up index (NDBI)^55^, urban index (UI)^56^ and visible red based built-up index (VRBI)^57^. We also included the normalized difference vegetation index (NDVI)^58^, the bare soil index (BSI)^59^, and the normalized burn ratio index (NBRI)^60^.

**Table 1:** Spectral indices used to model impervious cover across the four countries.

Spectral Index	Abbreviation	Equation	Source
Built-up area extraction index	BAEI	${baei}=\frac{{Red}+0.3}{{Green}+{Swir}1}$	^51^
Biophysical composition index	BCI	${bci}=\frac{{TCB}+{TCW}\div2-{TCG}}{{TCB}+{TCW}\div2+{TCG}}$	^52^
Band ratio for built-up area index	BRBA	${brba}=\frac{{Red}}{{Swir}1}$	^53^
Bare soil index	BSI	${bsi}=\frac{\left({Red}+{Swir}1\right)-({Nir}+{Blue})}{({Red}+{Swir}1)+({Nir}+{Blue})}$	^59^
Combinational biophysical composition index	CBCI	${cbci}=1.5* {mbsi}-{osavi}+0.5$	^54^
Normalized built-up area index	NBAI	${nbai}=\frac{({Swir}2-{Swir}1)/{Green}}{({Swir}2+{Swir}1)/{Green}}$	^53^
Normalized burned ratio index	NBRI	${nbri}=\frac{{Nir}-{Swir}1}{{Nir}+{Swir}1}$	^60^
Normalized difference built-up index	NDBI	${ndbi}=\frac{{Swir}1-{Nir}}{{Swir}1+{Nir}}$	^55^
Normalized difference vegetation index	NDVI	${ndvi}=\frac{{Nir}-{Red}}{{Nir}+{Red}}$	^58^
Urban index	UI	${uid}=\frac{{Swir}2-{Nir}}{{Swir}2+{Nir}}$	^56^
Visible red-based built-up index	VRBI	${vrbi}=\frac{{Red}-{Nir}}{{Red}+{Nir}}$	^57^

Tasseled cap indices, including brightness index (TCB), tasseled cap wetness index (TCW), tasseled cap greenness index (TCG), were used to generate biophysical composition index (BCI). Modified bare soil index (MBSI) and optimized soil adjusted vegetation index (OSAVI) were used to generate CBCI.

These indices were computed for all cloud-free pixels in all available Landsat images. Then, annual temporal metrics were generated for each index by summarizing across all cloud-free observations in a year^61,62^. These metrics were based on percentiles ranging from 5 to 95 at 5% intervals. We manually selected the best percentile for each spectral index by systematically varying the thresholds and visually comparing the resulting metrics with high-resolution data for cities with known changes (Table 2). The spectral signature of impervious surfaces is relatively stable, whereas the spectral characteristics of vegetation and soils vary seasonally with climate. Therefore, annual composites of impervious surface indices were generated using low percentiles (mostly 5–15%) to identify pixels where these indices remained high throughout the entire year. Conversely, higher percentiles were used for NDVI to distinguish areas where greenness remained low for most of the year. The percentiles were also varied across ecoregions to account for geographic variation in the spectral characteristics of urban and non-urban areas.

**Table 2:** Percentiles used to generate annual compositions by major ecological zones.

Spectral Index	Eastern Guinea Forest/Nigeria Lowlands Forest	Guinea Forest Savannah	Western Sudan Savannah
BAEI	15	10	10
BCI	10	10	10
BRBA	5	5	5
BSI	25	10	10
CBCI	15	5	5
NBAI	5	5	5
NBRI	15	40	15
NDBI	5	5	5
NDVI	55	85	75
UI	10	5	5
VRBI	40	40	40

Spectral index codes are described in Table 1.

### Training and validation data

To ensure we had sufficient sample points in both urban and non-urban areas, we created random sample points within two strata: inside the Africapolis city boundaries^44^ and outside of Africapolis city boundaries. We also used three country strata consisting of Ghana, Togo and Benin combined, and Nigeria, and four ecological zone strata. We randomly selected 300 points for each combination of the three strata, resulting in 600 points for each ecological zone within each country. In Ghana, we selected a total of 1800 points, consisting of 900 within cities and 900 outside cities across three ecological zones. Likewise, in Benin and Togo, we selected a total of 1200 points, consisting of 600 within cities and 600 outside cities across two ecological zones. In Nigeria, we also selected 1200 total points consisting of 600 within cities and 600 outside cities across the Nigeria Lowlands Forest and Guinea Forest Savannah ecological zones. Because there were typically fewer years of historical images in the Western Sudanian Savannah ecological zone in Nigeria, we selected 350 points in each city strata, resulting in 700 points.

**Fig. 4:**
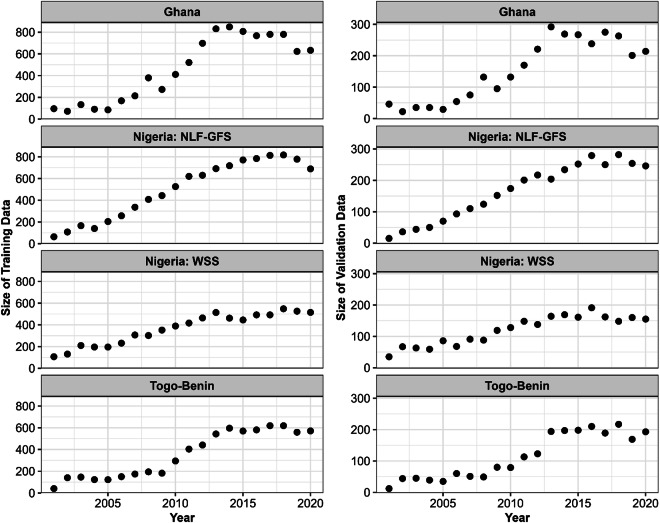
Temporal distribution of training and validation plots. The black dots are the total number of plots in each year. The Nigeria Lowlands Forest and Guinea Forest Savannah is NLF-GFS, and the Western Sudanian Savannah is WSS.

We generated a square of size 30 m for each sample location and overlaid a 5 × 5 grid of points. Each point on the grid represented a land cover of 4%. We overlaid these plots on all available very high-resolution (VHR) images in Google Earth Pro. There are geographical and temporal variations in the availability and quality of VHR images in Google Earth Pro. We collected training and validation for all years and locations where data were available and not obscured by clouds. We visually estimated the impervious cover as the number of points in each gridded polygon that covered impervious surfaces such as buildings, glass, concrete, and asphalt. The number of estimated points were multiplied by four to calculate the percent impervious cover for each plot. The total number of interpreted plots was 46,790, including 25,153 in Nigeria, 12,277 in Ghana, and 9,360 in Benin and Togo. We fitted the impervious surface models with 75% of the interpreted data and tested the accuracy using the remaining 25% as an independent validation dataset. The training and validation plots were distributed across all four countries from 2001–2020 (Fig. 4).

### Random forest regression

We used random forest regression to generate impervious cover using annual composites of spectral indices as predictors and percent impervious cover estimates from VHR as training data. Random forest is a machine learning algorithm that uses bootstrap sampling of the training data with random subsets of predictors to generate tree-based models and aggregates the results to generate ensemble predictions^32,63^. This approach decreases correlations among the trees and improves accuracy of predictions. Using the 35,231 training plots, we extracted the spectral index values corresponding to each year and sampled location. To reduce computational time, we trained four separate random forest models, including one in Ghana, one in Togo and Benin together, and two in Nigeria (one for the Nigeria Lowlands Forest and Guinea Forest Savannah ecological zones and one for the Western Sudanian Savannah ecological zone). We used the GEE smileRandom() function to fit each model with 1000 trees, 0.65 bag fraction, 6 predictors per split, 7 minimum leaf population, and output mode set to regression. These parameters were used to predict annual continuous impervious surface cover from 2001 to 2020.

### LandTrendr temporal segmentation

We applied LandTrendr to the annual impervious cover predictions from random forest to process the time series at individual pixel levels. We used the LandTrendr time series algorithm because it is less computationally demanding than CCDC and directly models annual change as a continuous variable, which was our desired output^35^. Although CCDC and BFAST offer the potential for monitoring sub-annual timing of changes^30,34^, previous studies of urban impervious surfaces show little to no variability across seasons^64,65^. Thus, LandTrendr was better suited for our application because it uses annual composites, reducing computation time and filling data gaps.

LandTrendr uses a temporal segmentation approach that fits linear regression models to annual values for each pixel with breakpoints and segments representing change history^35–37^. We used the fitted values from the segmented regression models generated by LandTrendr as our final estimated values of percent impervious cover. The reason for this step was to reduce noise in the random forest predictions of impervious cover and generate interpolated estimates for missing data points. Although the LandTrendr algorithm is designed to capture rapid declines in vegetation indices resulting from ecological disturbance, we adapted it to model increases in impervious cover resulting from urban expansion. We did this by reversing the numerical order of the impervious cover values so that zero represented 100% cover and hundred represented 0% cover. To accomplish this conversion, the random forest predictions of impervious cover were subtracted from 100, the LandTrendr algorithm was applied to these transformed data, and the resulting fitted values were subtracted from 100 to return them to their original order. We selected the best-fit parameters to run LandTrendr for our study area by conducting a sensitivity analysis, starting with the default parameters and systematically adjusting them by visually inspecting locations with known change histories (Table 3).

**Table 3:** LandTrendr segmentation parameters to process random forest impervious surface cover.

LandTrendr Parameters	Default Parameters	Study Parameters
maxSegments	6	6
spikeThreshold	0.9	0.75
vertexCountOvershoot	3	2
PreventOneYearRecovery	TRUE	TRUE
recoveryThreshold	0.25	0.01
pvalThreshold	0.05	0.05
bestModelProportion	0.75	0.5
minObservationsNeeded	6	6

### Developed area classification

**Fig. 5:**
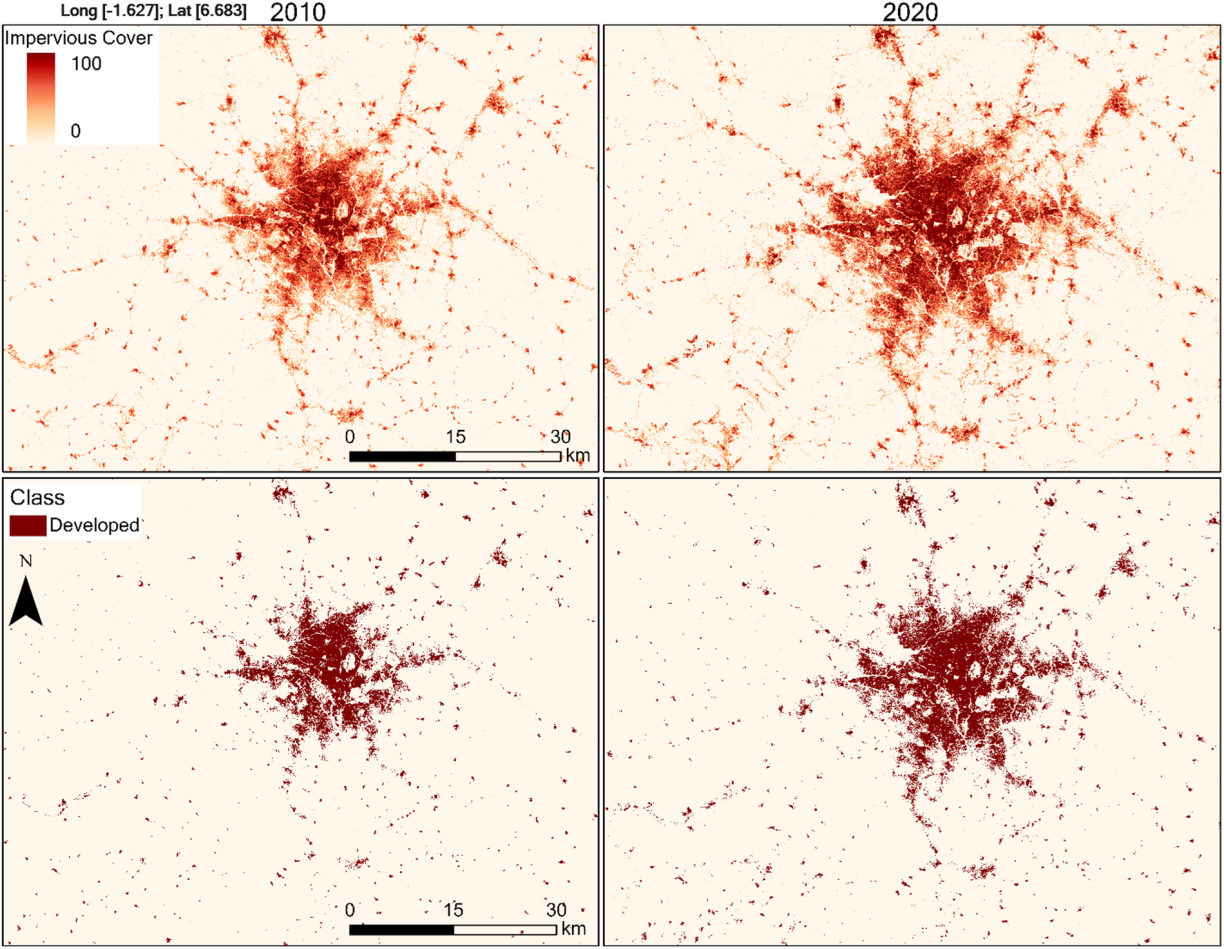
Continuous impervious surface cover and developed area classification for Kumasi and surrounding smaller cities in the southern portion of Ghana. The top row shows the continuous data, and the bottom row shows the classified data.

We classified LandTrendr fitted continuous impervious cover into developed and undeveloped. Impervious surface cover pixels values greater than 20% were classified as developed, and pixels less than or equal to 20% as undeveloped. We selected this threshold based on visual comparison of different thresholds with very high-resolution imagery. We applied a no-loss rule, which states that pixels remained developed even if the impervious surface cover for preceding years dropped below 20%. Similar rules are commonly used in urban land cover analysis to reduce false changes since impervious surfaces are mostly permanent^40,41^. We have provided an example for the continuous and developed area classification for Kumasi, Ghana, one of the cities in the study area with rapid urban expansion (Fig. 5).

### Model validation

We used 11,559 independent observations to evaluate impervious surface cover predictions from random forest and LandTrendr segmentation. We measured the association between observations and predictions using the predicted-observed r^2^. We measured prediction bias with mean error and quantified differences between observations and predictions using mean absolute error and root mean squared error. For the classification of developed areas, we computed a standard confusion matrix with overall accuracy, user’s and producer’s accuracies, and commission and omission errors.

Additionally, we used validation data for 2001–2018 to compare the accuracy of WADISC with existing global datasets with similar temporal and spatial resolution, consisting of global artificial impervious area (GAIA)^26^ and global impervious surface area (GISA)^66^. GAIA and GISA have binary classes consisting of impervious and non-impervious at 30 m resolution. GISA is available from 1972–2019, and GAIA has annual data from 1985–2018. We computed the average accuracies and errors for only these eighteen years because these are when data were available in all the impervious datasets we examined. We also compared the total developed area extents from these datasets across the four ecological zones: Eastern Guinea Forest (EGF), Guinea Forest Savannah (GFS), Nigeria Lowlands Forest (NLF) and Western Sudanian Savannah (WSS).

## Data Records

The West Africa Dataset of Impervious Surface Change (WADISC) comprises 30-m resolution continuous impervious cover and classified developed area datasets and is publicly accessible through Figshare: 10.6084/m9.figshare.24716481.v3^67^. The dataset consists of 40 Tiff files, including 20 continuous and 20 binary classifications, each containing developed area information across the four countries. The continuous impervious cover pixel values are percentages from 0 to 100, and the classified developed area values are 1 and 0, indicating presence or absence (Table 4).

**Table 4:** Details of impervious cover and developed area data.

Data	Spatial Extent	Temporal Resolution	Spatial Resolution	Coordinate System	Format	Data Type	Pixel values
Impervious Cover	Ghana, Togo, Benin, Nigeria	Annual	0.00027° × 0.00027°	WGS 1984 (ESPG: 4326)	TIFF	8 Bit unsigned integer	0–100
Developed	Ghana, Togo, Benin, Nigeria	Annual	0.00027° × 0.00027°	WGS 1984 (ESPG: 4326)	TIFF	8 Bit unsigned integer	0: Undeveloped 1: Developed

## Technical Validation

**Fig. 6:**
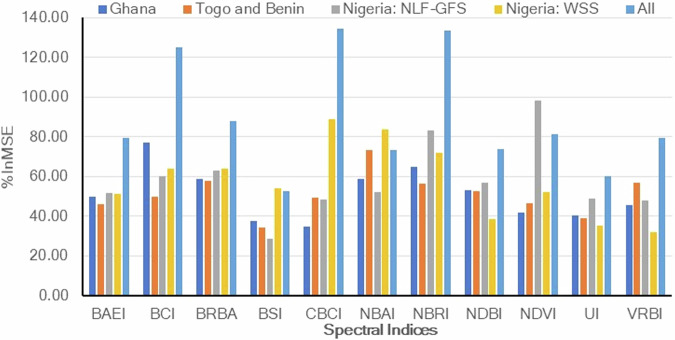
Importance of spectral indices in random forest predictions using percent increase in mean squared error (%InMSE). The % increase in mean squared error represents the increase in prediction errors (out-of-bag) resulting from the permutation of a random predictor. Spectral index codes are described in Table 1.

We used all 11 spectral indices to generate random forest impervious cover estimates because models using all the predictors had higher accuracies than those based on subsets. This finding was consistent with previous assessments of land cover change models that found models with more predictors had lower errors^50^. Across the four random forest models employed in this study, the relative importance of spectral indices varied, with BCI the most important predictor of impervious surface cover in Ghana, NBAI in Benin and Togo, NDVI in Nigeria Lowlands Forest and Guinea Forest Savannah in Nigeria, and CBCI in Western Sudanian Savannah in Nigeria (Fig. 6). BSI had relatively low importance, suggesting that the urban indices effectively discriminated impervious surfaces from soils.

**Fig. 7:**
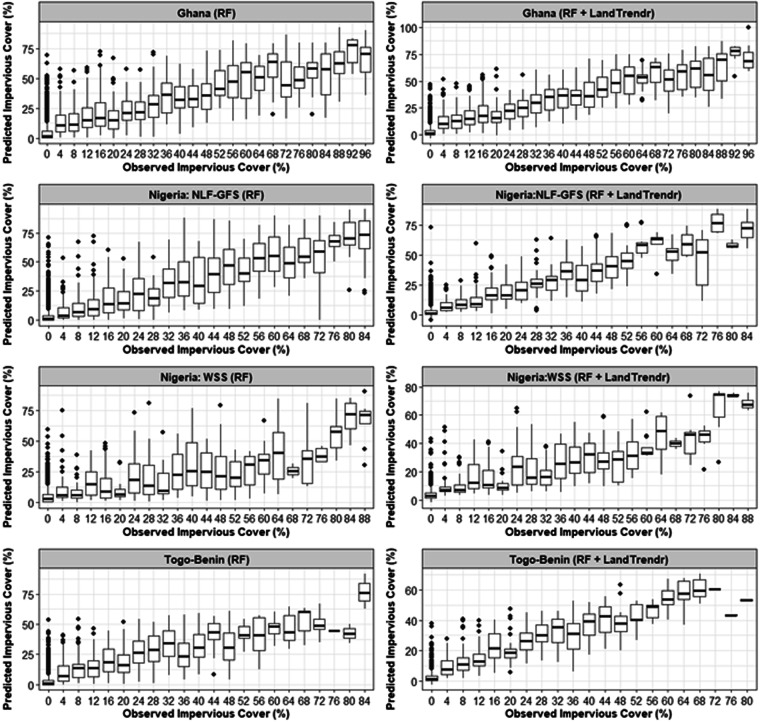
Associations between observed and predicted impervious surface cover estimates from Random Forest (RF) and processed LandTrendr. The outliers are the individual points, the 25^th^ percentile is the lower box edges, the 75^th^ percentile is the upper box edges, and the 50% percentile is the line separating the lower and upper boxes.

Using LandTrendr for temporal segmentation of the random forest predictions of impervious cover resulted in higher accuracies than predictions based on random forests alone (Table 5). The high predicted-observed r^2^ values indicated a strong linear association between the predictions and observations of impervious cover in the validation dataset. The strength of this association can also be seen in scatterplots of the predicted-observed relationship (Fig. 7). The magnitude of the mean error was close to zero when summarized across the entire study area, indicating that the predictions were unbiased. The overall mean absolute error was less than 6% cover and the root mean squared error was less than 10% cover, giving us confidence that the predictions can effectively distinguish areas with high versus low impervious cover.

**Table 5:** Accuracy for Random Forest and LandTrendr fitted continuous impervious surface cover.

Country	Metric	Random Forest	Random Forest + LandTrendr
Ghana	Predicted-Observed r²	0.67	0.79
	Mean Error	0.22	−0.30
	Mean Absolute Error	8.19	6.73
	Root Mean Squared Error	13.40	11.03
Togo and Benin	Predicted-Observed r²	0.62	0.81
	Mean Error	0.38	0.44
	Mean Absolute Error	6.14	4.78
	Root Mean Squared Error	10.89	8.01
Nigeria: Nigeria Lowlands Forest and Guinea Forest Savannah	Predicted-Observed r²	0.71	0.81
	Mean Error	0.46	0.34
	Mean Absolute Error	6.59	4.36
	Root Mean Squared Error	12.17	8.05
Nigeria: Western Sudan Savannah	Predicted-Observed r²	0.70	0.82
	Mean Error	0.13	−0.47
	Mean Absolute Error	8.62	6.88
	Root Mean Squared Error	14.53	11.65
All	Predicted-Observed r²	0.60	0.81
	Mean Error	0.28	−0.03
	Mean Absolute Error	7.81	5.73
	Root Mean Squared Error	13.45	9.93

The overall accuracy of the developed area classification was high, with a value of 93% across the entire study area, and 90% in Ghana, 94% in Togo-Benin, 96% in Nigeria Lowlands Forest and Guinea Forest Savannah, and 93% in Nigeria Western Sudan Savannah (Table 6). Class-level accuracy statistics were generally higher for non-developed areas than for developed areas. However, the overall producer’s and user’s accuracies for developed areas were still relatively high at ~80%. The errors of omission and commission had similar magnitudes for each class, indicating that there was not a strong bias toward overpredicting or underpredicting developed areas.

**Table 6:** Confusion matrix of developed and undeveloped areas classifications from 2001–2020.

	Observation					
	Country	Class	Undeveloped	Developed	User	Commission
**Prediction**	Ghana	Undeveloped	2210	154	0.93.49	6.51
		Developed	165	534	76.39	23.61
		Producer	93.05	77.62	**89.59**	
		Omission	6.95	22.38		***10.41***
	Togo and Benin	Undeveloped	1868	63	96.74	3.26
		Developed	84	282	77.05	22.95
		Producer	95.70	81.74	**93.60**	
		Omission	4.30	18.26		***6.40***
	Nigeria: Nigeria Lowlands & Guinea Forest Savannah	Undeveloped	2910	74	97.52	2.48
		Developed	79	396	83.37	16.63
		Producer	97.36	84.26	**95.58**	
		Omission	2.64	15.74		***4.42***
	Nigeria: Western Sudan Savannah	Undeveloped	2175	134	94.20	5.80
		Developed	73	398	84.50	15.50
		Producer	96.75	74.81	**92.55**	
		Omission	3.25	25.19		***7.45***
	All	Undeveloped	9163	425	95.57	4.43
		Developed	401	1610	80.06	19.94
		Producer	95.81	79.12	**92.88**	
		Omission	4.19	20.88		***7.12***

The overall accuracy of the data is in bold, and the misclassified error is in bold italics.

### Comparison with global impervious data

The average overall accuracy from 2001 to 2018 was highest in WADISC with 93.16%, whereas GISA was 90.25%, and GAIA was 89.28% (Table 7). Also, the omission error, representing the probability of misclassifying developed areas in the reference data, was 19.23% in WADISC but increased to 24.91% in GISA and 31.56% in GAIA. The commission error, indicating the probability of misclassifying undeveloped areas, was 21.27% in WADISC but increased considerably to 30.92% in GISA and 32.38 in GAIA. In addition, the F-score representing the harmonic mean between the producer and user’s accuracy was highest for WADISC followed by GISA and GAIA.

**Table 7:** Accuracy comparison with global impervious datasets from 2001–2018.

Observation							
	Dataset	Class	Undeveloped	Developed	User	Commission	F-score
Prediction	WADISC	Undeveloped	7909	318	96.13	3.87	
		Developed	361	1336	78.73	21.27	0.7974
		Producer	95.63	80.77	**93.16**		
		Omission	4.37	19.23		***6.84***	
	GISA	Undeveloped	7714	412	94.93	5.07	
		Developed	556	1242	69.08	30.92	0.7196
		Producer	93.28	75.09	**90.25**		
		Omission	6.72	24.91		***9.75***	
	GAIA	Undeveloped	7728	522	93.67	6.33	
		Developed	542	1132	67.62	32.38	0.6803
		Producer	93.45	68.44	**89.28**		
		Omission	6.55	31.56		***10.72***	

The overall accuracy of the data is in bold, and the misclassified error is in bold italics.

**Fig. 8:**
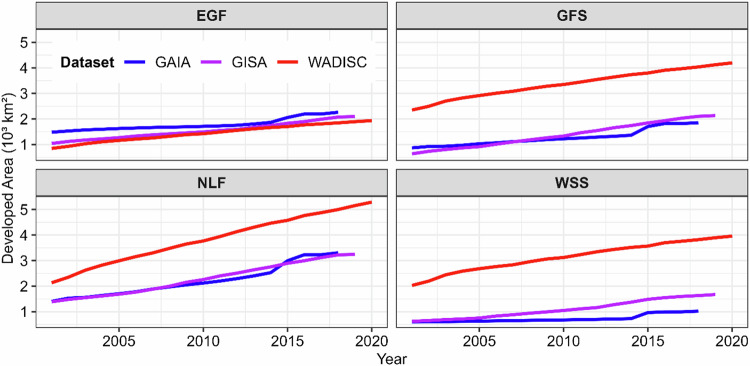
Developed area of the GAIA and GISA datasets compared to WADISC across ecological zones in the study area. The red line shows the WADISC developed area from 2001–2020, the blue line shows the GAIA developed area from 2001–2018, and the purple line shows the GISA developed area from 2001–2019. The ecological zones are Eastern Guinea Forest (EGF), Guinea Forest Savannah (GFS), Nigeria Lowlands Forest (NLF), and Western Sudanian Savannah (WSS).

**Fig. 9:**
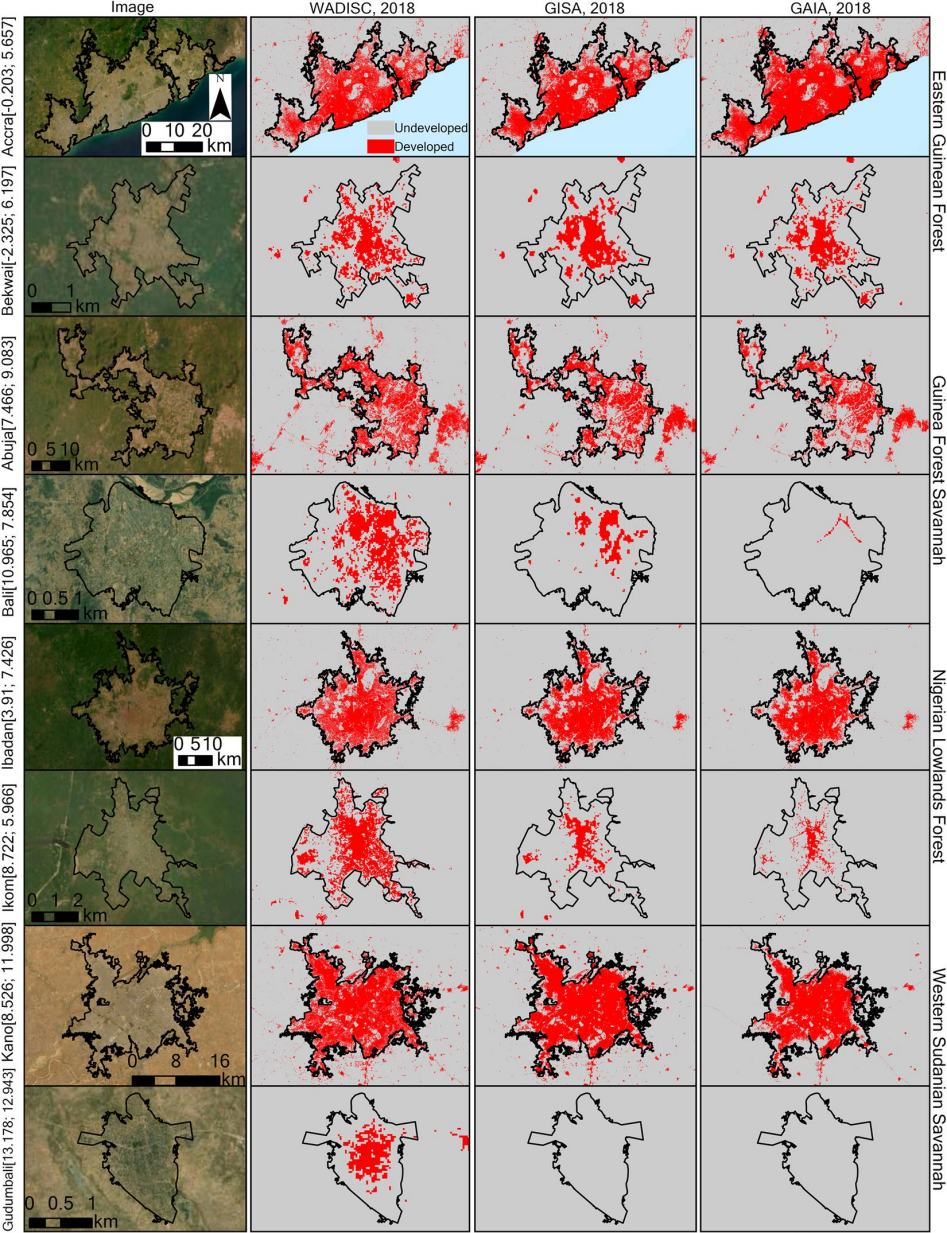
Comparison across cities in different ecological zones. The primary cities with over one million urban populations are Accra, Abuja, Ibadan, and Kano, and the smaller cities with between ten thousand and one million population are Bekwai, Bali, Ikom, and Gudumbali. The numbers in parenthesis are the longitudes and latitudes.

The developed area comparison across the various datasets shows that GISA and GAIA were similar, and WADISC was higher (Fig. 8). Examples of developed area in primary cities with over a million population and secondary cities with between ten thousand and one million population in different ecological zones are shown in Fig. 9. Developed area extent in all three datasets were similar in the Eastern Guinea Forest (EGF). The developed area for WADISC, however, was higher than GAIA and GISA in the Guinea Forest Savannah (GFS), Nigeria Lowlands Forest (NLF), and Western Sudanian Savannah (WSS). Our findings are consistent with previous studies that indicate most existing global datasets underestimate urban extent in the study area^1,11^.

The global datasets we examined have longer temporal extents, allowing for historical developed area analysis as far back as 1972 for GAIA and 1985 for GISA. Although WADISC covers only four countries, it encompasses the majority of cities in West Africa, and it is the first annual regional impervious cover dataset with higher accuracies and lower errors than existing global datasets. The accuracy of WADISC was also high even in the Western Sudanian Savannah ecological zone with semi-arid conditions where urban areas are often difficult to classify with remote sensing data^1^. The WADISC calibration accounted for sub-regional ecological differences in urban morphology and background conditions, resulting in higher accuracy and better detection of developed areas than the global datasets (Fig. 9). Additionally, the WADISC data captured smaller settlements that are often excluded or misclassified in global datasets (Fig. 9).

## Usage Notes

### Change analysis

We demonstrated the potential uses of this data by quantifying and comparing the rates of developed area changes across countries and comparing the patterns of developed area changes for selected cities. The total developed area in each country more than doubled between 2001 and 2020, with a 2.2-fold increase in Benin, 2.3 in Ghana, 2.4 in Nigeria, and 2.1 in Togo (Table 8). Thus, Nigeria had the highest annual increase in developed areas, followed by Ghana, Benin, and Togo.

**Table 8:** Total developed area and percentage annual expansion rate from 2001–2020.

Country	2001	2020	Change
	Developed Area (km^2^)	Developed Area (km^2^)	Annual Expansion rate (%)
Benin	217.52	474.33	4.19
Ghana	690.75	1583.14	4.46
Nigeria	2931.72	7012.58	4.70
Togo	160.75	331.77	3.89
All	4001.73	9401.81	4.60

**Fig. 10:**
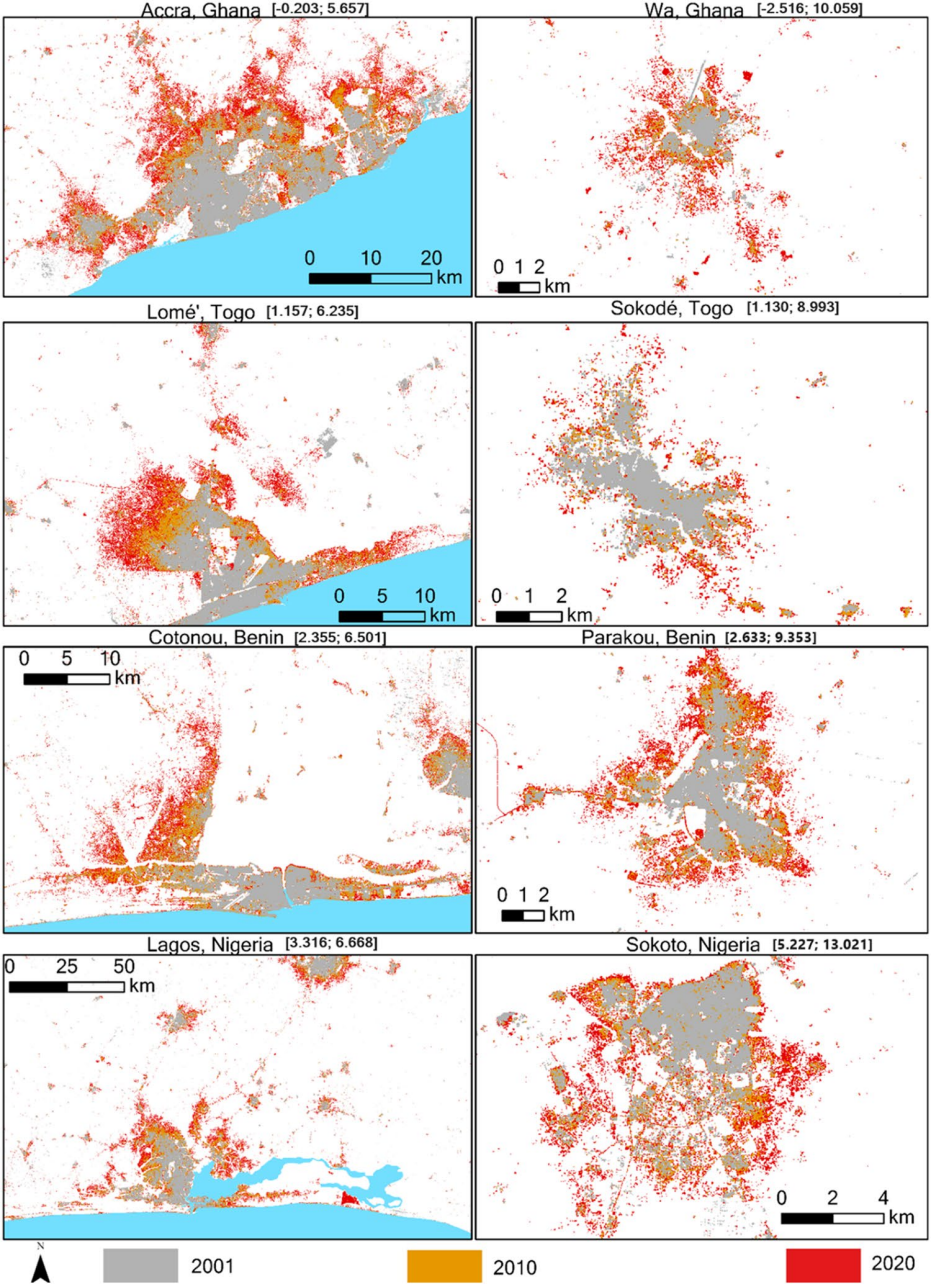
Change in developed area for subset of cities in each country for three-time points. The gray shows the initially developed area in 2001, the orange shows the developed area in 2010, and the red shows the developed area in 2020. The numbers in parenthesis are the longitudes and latitudes of each city, respectively.

Maps of the developed area extent for the largest primary city and secondary city each in Ghana, Togo, Benin, and Nigeria with rapid urban growth shows that new developed areas rapidly filled open spaces and expanded outwards from the initial developed area (Fig. 10). A recent study used WADISC and quantified the expansion patterns across cities of varying population sizes, and found cities mostly sprawled faster than infilled, especially in smaller secondary cities^43^. Different growth patterns are influenced by differences in national urban development plans, rates of urbanization, land tenure systems, and foreign direct investments. The sprawling outward expansion and high expansion rates are challenging to manage due to limited resources. Although urban expansion occurs locally, the impacts are much broader, meaning regional-level analysis can better inform sustainable development decisions.

WADISC can support research and assessment of urban expansion impact on heat stress^68,69^, urban malaria incidence^70–72^, greenhouse gas emission^73,74^, urban floods^75^, cropland loss^76,77^, biodiversity loss and habitat fragmentation^2,8^, within and across countries. For example, Van Vliet (2019) found urban expansion resulted in 122 million tons decrease in cereal production per year from 1992–2015. Also, urban areas are susceptible to increased risk of heat stress in the Sydney region^68^. These studies mostly found that urban expansion negatively impacts people and the environment. However, some studies found that compact urban expansion had a negative association with air pollution^78^ and energy and resource usage^79^ but was positively associated with economic productivity^80^. Thus, WADISC can support diverse research and policy assessments across multiple scales in Ghana, Togo, Benin, and Nigeria.

### Potential limitations

When classifying change in developed area, we applied a rule of no-developed area loss, which states that developed pixels are irreversible. This is a standard approach in land cover change analysis and had a relatively minor effect on the predictions of developed areas and their accuracy. There were only a few cities where this rule had a noticeable effect, correcting for false transitions of developed to non-developed areas that arose from noise in the spectral data. However, there are other areas, particularly in the northern portions of the study area, where climate fluctuations or conflict may result in abandonment of developed areas with a resulting transition to non-developed.

Additionally, all Landsat 7 data collected after May 31, 2003 have data gaps resulting from the scan line corrector (SLC) off. The scan line corrector removes the zigzag motion produced by across and along track movement of the sensor. Despite the SCL failure, these data are still geometrically and radiometrically correct and are measured at the same precision as prior to the anomaly. Although most SLC gaps are filled in the process of generating the annual temporal metrics, the process sometimes results in different values in the SLC gaps, particularly between 2003 and 2013 when only Landsat 7 images are available. These spatial anomalies in the temporal metrics influence the patterns of developed pixels in some locations. However, the overall accuracy of the continuous impervious surface estimates and developed area classification is still high.

We have provided access to the underlying continuous impervious surface data as well as the classified developed area data^67^. These data can be used to generate developed area classifications using different impervious surface thresholds and to analyze change using different types of transition rules. All land cover datasets contain some error, but our rigorous quantitative validation combined with visual assessment of the predicted changes indicate that the predictions of impervious surfaces and developed areas accurately capture their patterns and magnitudes of change. We have provided access to the underlying code for generating the data products in Google Earth Engine and GitHub, and users are thus able to conduct more detailed evaluations of the underlying techniques and potentially update and extend the methods to improve their accuracy.

## Data Availability

The scripts used to generate the impervious surface cover data are available and accessible in fighshare^67^. The scripts are available in Google Earth Engine: https://code.earthengine.google.com/?accept_repo=users/korahandrews/WADISC, and on GitHub through this link: https://github.com/Kora0003/WADISC.
